# Healthcare in the Modern Era: Launching a Telemedicine-Based OPD Consultation in Rural Pune (Process, Results, and Challenges)

**DOI:** 10.7759/cureus.60310

**Published:** 2024-05-14

**Authors:** Varsha Vaidya, Vaibhav Patil, Jitendra Oswal, Arvinder Narula, Yogesh Khare, Prajakta Patil, Ruma Deshpande, Snehal Lunge, Sukanya Dasgupta, Ramdas Dahiphale, Rohit Kulkarni, Amit Mahajan, Srinivasa Chelluri, Advait Teli

**Affiliations:** 1 Community Medicine, Bharati Vidyapeeth (Deemed to be University) Medical College, Pune, IND; 2 Cardiology, Bharati Vidyapeeth (Deemed to be University) Medical College, Pune, IND; 3 Pediatric Rheumatology, Bharati Vidyapeeth (Deemed to be University) Medical College, Pune, IND; 4 Internal Medicine, Bharati Vidyapeeth (Deemed to be University) Medical College, Pune, IND; 5 Pediatric Endocrinology, Bharati Vidyapeeth (Deemed to be University) Medical College, Pune, IND; 6 Dermatology, Bharati Vidyapeeth (Deemed to be University) Medical College, Pune, IND; 7 Pediatrics, Bharati Vidyapeeth (Deemed to be University) Medical College, Pune, IND; 8 Orthopaedics, Bharati Vidyapeeth (Deemed to be University) Medical College, Pune, IND; 9 Orthopaedics and Trauma, Bharati Hospital, Pune, IND

**Keywords:** tele-dermatology, telemedicine, barriers to use of telemedicine, tele-consultation, telemedicine patient satisfaction

## Abstract

Introduction

Telemedicine serves as a means of overcoming geographical barriers and increasing access to specialist care. This study focuses on the impact of telemedicine on the early diagnosis and treatment of patients, as well as its effect on patient satisfaction. In addition, the study examines the obstacles and facilitators that influence the implementation of telemedicine.

Objectives

The primary objectives of this study are to assess the effectiveness of telemedicine in facilitating early diagnosis and treatment for patients in need of specialist consultations, to evaluate patient satisfaction with specialist care delivered through telemedicine, and to identify the factors that influence the successful implementation of telemedicine in rural healthcare centers.

Methodology

An exploratory feasibility study was carried out at two rural health training centers (RHTCs) over a one-year period, enrolling 400 patients requiring specialist consultations. The study involved establishing a telemedicine center, implementing teleconsultations, and collecting data through patient interviews and self-administered questionnaires.

Results

A majority of teleconsultations, over 79%, were deemed valuable by medical officers, resulting in improved management, better counseling, and earlier diagnoses. More than 76% of patients found telemedicine to be acceptable due to the reduction in travel time and cost. The most common health concerns among patients were diabetes, hypertension, and skin disorders. The study also revealed several challenges, including limited specialist personnel, waiting times, prescription limitations, and connectivity issues.

Discussion

Telemedicine has proven to be a valuable tool for rural healthcare delivery, providing patients with access to specialist consultations and improving patient outcomes. Both patients and medical officers reported positive experiences with telemedicine. The findings of this study align with existing literature, which highlights the benefits of telemedicine in managing chronic diseases and increasing patient satisfaction. However, it is crucial to address challenges, such as personnel limitations and connectivity issues, to optimize telemedicine's effectiveness.

Conclusion

Telemedicine offers great potential for enhancing access to specialist care and achieving universal healthcare in rural areas. Despite its limitations, telemedicine demonstrates promising outcomes and warrants further development and optimization to ensure its successful implementation in rural healthcare centers.

## Introduction

The World Health Organization (WHO) characterizes telemedicine as the provision of healthcare services through the utilization of information and communication technologies, especially when distance is a critical factor [1]. Telemedicine has proven to be a valuable asset for patients, reducing the need for travel and connecting them with specialist physicians remotely. This approach not only decreases the number of undiagnosed and untimely diagnosed [2] but also increases patient satisfaction [3] and helps us achieve universal healthcare.

Telemedicine initiatives are playing a crucial role in eradicating geographical barriers and enhancing access to quality healthcare even in rural areas. Despite its potential, the widespread adoption of telemedicine is impeded by limited awareness and acceptance among both the general public and medical professionals.

In light of these challenges, our study endeavors to explore the perspectives of medical officers and specialist doctors on telemedicine, while assessing its effectiveness in early diagnosis, treatment, and patient satisfaction levels. In addition, we aim to identify the barriers and facilitators influencing the successful implementation of telemedicine in providing specialist access to healthcare centers.

## Materials and methods

An exploratory feasibility study was conducted at two rural health training centers (RHTCs) in India, namely, Rural Health Training Center, Lavale, and Rural Health Outpatient Department (OPD), Nasrapur, administered by the Departments of Community Medicine and Pediatrics in rural field practice areas. These RHTCs collectively cater to a population of 89,414 individuals. The study enrolled all patients requiring specialist consultation who attended outpatient departments (OPDs) or screening camps organized by the RHTCs within a one-year period.

Inclusion and exclusion criteria

The inclusion criteria included OPD patients attending RHTC OPD requiring specialist consultation and patients attending screening camps (center-based or community-based) organized by RHTC OPD who require specialist consultation. The exclusion criteria include patients attending OPD requiring urgent surgical intervention, as they could not benefit from teleconsultation.

Ethical consideration

The research project obtained approval from Bharati Vidyapeeth (Deemed to be University) Medical College, Pune, Institutional Ethics Committee before initiation. Informed consent was obtained from all participants, ensuring confidentiality and adherence to ethical principles. In addition, a memorandum of understanding was established with Evolko, India, to safeguard patient data confidentiality.

Establishment of a telemedicine center and data collection

The study was divided into three phases: Phase I is the establishment of telemedicine consultation center at RHTCs, which includes the following steps: a) software configuration and installation of digital devices for vital sign measurements, b) utilization of an Android tablet (version 8.0 or higher) for patient information and prescriptions, c) installation of WiFi-enabled printers at clinic locations, d) training of study investigators on software usage, and e) establishment of a telemedicine center at a tertiary care hospital with 11 contributing physicians.

Phase II is the implementation of telemedicine consultancy (one year), which includes a) recording basic patient details, chief complaints, and examination results in software; b) real-time assessments by a specialist from Bharati Hospital; c) video calls for additional information if necessary; d) specialist recommendations for further examinations and treatment plans; and e) dispensing of prescriptions and counseling at the outpatient department, followed by scheduling of follow-up appointments at RHTCs.

Phase III is the data analysis, compilation, and interpretation, which includes a) collection of digital data including demographic details, medical history, examination results, investigation findings, consultation details, medication prescriptions, treatment outcomes, and follow-up information; b) assessment of patient satisfaction and perceived advantages and limitations of telemedicine through structured interviews conducted in Marathi; c) evaluation of effectiveness, barriers, facilitators, advantages, and limitations of teleconsultation through self-administered structured questionnaires in English; and d) data analysis conducted weekly and compiled using IBM SPSS Statistics for Windows, Version 25.0 (released 2017, IBM Corp., Armonk, NY), with outcomes presented in tabular and graphical formats.

## Results

The project commenced in August 2021 and concluded in July 2022, during which a cohort of 400 patients requiring specialist consultation participated. Of these patients, more than 59.9% were aged 46 years or older. Table 1 illustrates the demographic breakdown of the patient population. Common health concerns prompting referral included diabetes, hypertension, joint pain, skin disorders, fever, cough, cold, and malnutrition in pediatric cases.

**Table 1:** Demographic details of the patients

Demographic		Frequency (%)
Age (in years)	≤5	37 (10.2)
	6 to 15	20 (5.5)
	16 to19	4 (1.1)
	20 to 45	85 (23.4)
	46 to 60	81 (22.3)
	>60	137 (37.6)
Gender	Female	188 (51.6)
	Male	176 (48.4)

A total of 364 patients of the 400 enrolled underwent triage assessment, as 36 encountered network issues, necessitating consultations via phone and WhatsApp. Chief complaints of referred patients are summarized in Table 2.

**Table 2:** Chief complaints of referred patients

Chief complaint	N (%)
Diabetes	98 (26.92)
Joint pain and other orthopedics conditions	54 (14.84)
Pediatrics	52 (14.29)
Skin-related complaints	35 (9.62)
Hypertension	34 (9.34)
Others	16 (4.40)
Weakness	13 (3.57)
Cough/cold	10 (2.75)
Urinary tract infection	10 (2.75)
Breathlessness	9 (2.47)
Fever	8 (2.20)
Allergies	7 (1.92)
Pain in the abdomen	7 (1.92)
Chest pain	4 (1.10)
PV bleeding	4 (1.10)
Antenatal care	3 (0.82)
Total	364 (100)

Table 3 delineates specialty-wise referrals for teleconsultation, while Table 4 presents age and gender distributions of patients with diabetes mellitus, hypertension, and skin diseases. It was perceived by the medical officer in RHTC that these patients had better HbA1c and blood pressure control due to specialist consultation.

**Table 3:** Speciality-wise referrals done

Referred specialty	Frequency (%)
Cardiology	116 (31.9)
General medicine	123 (33.8)
Pediatrics	52 (14.3)
Dermatology	35 (9.6)
Obstetrics & gynecology	18 (4.9)
Orthopedics	17 (4.7)
Medical officer RHTC	3 (0.8)
Total	364 (100)

Teleconsultations proved highly valuable in 288 out of the 364 referrals (79.1%), per medical officers' assessments. This value primarily manifested in improved management (56.9%), enhanced counseling (17.0%), confirmation of diagnoses, and recommendations for more effective investigations and earlier diagnoses (4.1%). Over 76.1% of patients found telemedicine acceptable and beneficial in terms of reduced travel, time, and costs.

Table 4 and Table 5 summarize medical officers' and patients' perceptions of telemedicine, with multiple choices selected by participants. Noteworthy obstacles to project execution included the shortage of specialized personnel, prolonged waiting times, inability to dispense prescriptions, and connectivity issues.

**Table 4:** Medical officer perception about telemedicine* *Multiple choices selected by the participants.

Medical officer perceptions about telemedicine	Frequency (%)
Consultancy added value	288 (79.1)
Better management	207 (56.9)
Better counselling	62 (17.0)
Early diagnosis	15 (4.1)
Suggesting better investigation	17 (4.7)
Confirming medical officers' diagnosis	21 (5.8)

**Table 5:** Patients’ perception about telemedicine* *Multiple choices selected by the participants.

Patients Perceptions about telemedicine	Frequency (%)
Telemedicine felt acceptable	277 (76.1)
Ready to pay for telemedicine services	242 (66.5)
Saves time	281 (77.2)
Find it convenient	277 (76.1)

The limitations of telemedicine consultations faced during the study are highlighted in Table 6.

**Table 6:** Limitations faced during telemedicine consults *Multiple choices selected by the participants.

Limitations faced during telemedicine consults	Frequency (%)
Prescription dispensation issues	44 (12.1)
Network-related issues	42 (11.5)
Language issues	35 (9.6)
Specialist unavailability	9 (2.5)

Notably, a case of undescended testis was diagnosed early and thus managed effectively. Similarly, a case of complete heart block was diagnosed early and then immediately referred for surgical management to a tertiary care center.

## Discussion

Telemedicine has emerged as a vital tool in modern healthcare, offering innovative solutions to bridge the gap between specialist care and patients in remote areas. The present study reinforces the growing body of evidence supporting telemedicine's effectiveness in providing specialist consultations, particularly for geographically isolated patients. With over 59.9% of patients aged 46 years or older benefiting from teleconsultations, it is evident that telemedicine plays a crucial role in addressing the healthcare needs of diverse age groups. The distribution of patients based on chief complaints and specialty-wise referrals further emphasizes the relevance of telemedicine in facilitating access to specialized care for a wide range of health concerns. Comparing these findings with existing literature, studies by Bashshur et al. (2016) [2] and Hilty et al. (2013) [4] have also highlighted the positive impact of telemedicine interventions on reducing hospital admissions, improving patient outcomes, and providing comprehensive care to the patient.

Patient and medical officer perceptions toward telemedicine are crucial factors in determining the acceptability and effectiveness of teleconsultations. In our study, we found that telemedicine has been met with positive perceptions from both patients and medical professionals, especially in the context of better management, counseling, time-saving, and convenience. These viewpoints align with the findings of Hailey et al. (2019) [5], which reported high patient satisfaction with telemedicine and emphasized the growing acceptance and benefits of telemedicine in healthcare delivery. Studies have shown that despite physical separation, consultation via telemedicine is not inferior to in-person consultations, with patients reporting similar satisfaction [6]. Telemedicine enables one to get multispecialty interactive teleconsultations, which would have been nearly impossible for those living in rural and hard-to-reach areas. Such means of communication reduce the out-of-pocket expenditure faced by the patient, especially in terms of travel, which can pose as a hurdle in delivering healthcare. As our study was conducted during the COVID-19 pandemic, telemedicine proved to be invaluable in facilitating patient access to specialists amid movement restrictions and safety concerns.

It was also noted that patients with chronic illnesses, such as diabetes and hypertension, were better managed with the help of telemedicine. The IDEATel study on diabetes management through telemedicine showed improvement in the HbA1c, LDL-cholesterol, and blood pressure levels [7]. As the diabetic capital of the world and a majority of the diabetics live in rural India, it is of paramount importance to achieve cost-effective ways to manage these patients, which has been seen with the help of telemedicine [8]. Noncompliance with medication still remains a public health concern in the rural parts of India, which could lead to multi-organ failure. Studies have shown that telemedicine consults are able to screen diabetic retinopathy, one of the earliest markers of failure [9]. Such a comprehensive screening is difficult for a general practitioner in rural India due to the lack of resources and skills. Similar to our study, it has been observed that patients with hypertension had better blood pressure control and medication adherence [10,11]. Thus, such chronic illnesses can be effectively managed, and it can be possible to provide comprehensive care in rural and hard-to-reach places.

We also noticed the demand for teledermatology in our study where people with various skin lesions were connected with dermatologists, which improved the quality of diagnosis and management. The lack of dermatologists in rural areas leaves various skin lesions and patients' problems unaddressed [12,13]. Thus, telemedicine could help deliver universal health coverage to all.

Despite these benefits, our study also identified significant challenges, including the lack of specialized personnel (2.5%), extended waiting times for specialist consults (though still notably better than the extensive travel times required), prescription dispensation issues (12.1%), connectivity difficulties (11.5%), and language barriers (9.6%). These obstacles are consistent with the findings of Wade et al. [14] (2014) and Hadian et al. [15] (2024), who highlighted similar barriers to telemedicine adoption. Addressing these challenges is essential for optimizing the effectiveness of telemedicine initiatives and ensuring seamless healthcare delivery.

## Conclusions

Although there remain some drawbacks of telemedicine and telecare, they are associated with better patient outcomes and satisfaction. Telemedicine shows promise in improving the patient-to-specialist connect, which was seemingly impossible in the rural and hard-to-reach parts of India. Thus, the adaptation of telemedicine can help us provide universal comprehensive healthcare to all.
